# Natural reference structures for three-dimensional maxillary regional superimposition in growing patients

**DOI:** 10.1186/s12903-023-03367-3

**Published:** 2023-09-08

**Authors:** Yi Fan, Bing Han, Yungeng Zhang, Yixiao Guo, Wei Li, Huanhuan Chen, Chenda Meng, Anthony Penington, Paul Schneider, Yuru Pei, Gui Chen, Tianmin Xu

**Affiliations:** 1 Third Clinical Division, Peking University School and Hospital of Stomatology, Beijing, China; 2 Department of Orthodontics, Peking University School and Hospital of Stomatology, Beijing, China; 3 National Engineering Laboratory for Digital and Material Technology of Stomatology, Beijing Key Laboratory of Digital Stomatology, Peking University School and Hospital of Stomatology, Beijing, China; 4 Key Laboratory of Machine Perception (MOE), Department of Machine Intelligence, School of Artificial Intelligence and Technology, Peking University, Beijing, China; 5 Department of Paediatrics, The University of Melbourne, Royal Children’s Hospital, Melbourne, Australia; 6 Facial Science, Murdoch Children’s Research Institute, Melbourne, Australia; 7 Melbourne Dental School, The University of Melbourne, Melbourne, Australia

**Keywords:** Maxillary superimposition, Growing children, Image registration, Stable regions

## Abstract

**Background:**

Assessment of growth-related or treatment-related changes in the maxilla requires a reliable method of superimposition. Such methods are well established for two-dimensional (2D) cephalometric images but not yet for three-dimensions (3D). The aims of this study were to identify natural reference structures (NRS) for the maxilla in growing patients in 3D, opportunistically using orthodontic mini-screws as reference; and to test the applicability of the proposed NRS for maxillary superimposition by assessing the concordance of this approach with Björk’s ‘stable reference structures’ in lateral projection.

**Methods:**

The stability of the mini-screws was tested on longitudinal pairs of pre- and post-orthodontic cone-beam computed tomography (CBCT) images by measuring the distance changes between screws. After verifying the stability of the mini-screws, rigid registration was performed for aligning the stable mini-screws. Then, non-rigid registration was used to establish the dense voxel-correspondence among CBCT images and calculate the displacement of each voxel belonging to the maxilla relative to the mini-screws. The displacement vectors were transformed to a standardized maxillary template to categorize the stability of the internal structures statistically. Those voxels that displaced less relative to the mini-screws were considered as the natural reference structures (NRS) for the maxilla. Test samples included another dataset of longitudinal CBCT scans. They were used to evaluate the applicability of the proposed NRS for maxillary superimposition. We assessed whether aligning the maxilla with proposed NRS is in concordance with the maxillary internal reference structures superimposition in the traditional 2D lateral view as suggested by Björk. This was quantitively assessed by comparing the mean sagittal and vertical tooth movements for both superimposition methods.

**Results:**

The stability of the mini-screws was tested on 10 pairs of pre- and post-orthodontic cone-beam computed tomography (CBCT) images (T1: 12.9 ± 0.8 yrs, T2: 14.8 ± 0.7 yrs). Both the loaded and the unloaded mini-screws were shown to be stable during orthodontic treatment, which indicates that they can be used as reference points. By analyzing the deformation map of the maxilla, we confirmed that the infraorbital rims, maxilla around the piriform foramen, the infrazygomatic crest and the hard palate (palatal vault more than  1 cm distal to incisor foramen except the palatal suture) were stable during growth. Another dataset of longitudinal CBCT scans (T1: 12.2 ± 0.63 yrs, T2: 15.2 ± 0.96 yrs) was used to assess the concordance of this approach with Björk’s ‘stable reference structures’. The movement of the maxillary first molar and central incisor showed no statistically significant difference when superimposing the test images with the proposed NRS or with the classic Björk maxillary superimposition in the lateral view.

**Conclusions:**

The infraorbital rims, maxilla around the piriform foramen, the infrazygomatic crest and the hard palate (palatal vault more than 1 cm posterior to incisal foramen except the palatal suture) were identified as stable regions in the maxilla. These stable structures can be used for maxillary superimposition in 3D and generate comparable results to Björk superimposition in the lateral view.

## Introduction

Evaluation of tooth movement and assessment of craniofacial growth are topics of great importance in Orthodontics. Maxillary growth and treatment changes in the maxillary dentoalveolar complex can be measured in a biologically meaningful way by superimposing images taken at two or more separate time points. Image registration, also known as superimposition, is the process of overlaying images (two or more) of the same scene taken at different times or in different treatment stages into the same coordinate so that differences between the images can be interpreted. Methods of superimposition of the maxilla are well established for two-dimensional (2D) cephalometric images but not yet for three-dimensional (3D) volumes [1].

Superimposition of the maxilla has traditionally been achieved by identifying regions which are relatively stable during the period of interest. In the twentieth century, Dr. Arne Björk et al. combined the methods of serial lateral cephalograms with metallic implantation to describe jaw growth. They discovered that the only stable structure in the maxilla is the anterior contour of the zygomatic process as this contour kept a constant relation to implants in the infrazygomatic crest and closely followed the natural growth rotation of the maxilla [2]. Superimposition of two maxillae can therefore be achieved in the absence of implants by rotating and translating one cephalogram to another until the cumulative distance between the stable region is minimized. As the orbits do not increase in height from childhood through adolescence to the same degree as the nasal cavity, the proportion of the apposition of the orbits and the resorption of the nasal floor have also been used to assist maxillary regional superimposition [2]. To date, this method remains recommended by the American Board of Orthodontics for maxillary superimposition in 2D cephalograms. Most knowledge concerning jaw growth and orthodontic treatment effects is derived from this superimposition approach.

Recently, computed tomography (CT) and cone-beam computed tomography (CBCT) have become popular diagnostic tools in dentistry. These technologies represent craniofacial structures as complex three-dimensional objects and contain much more information about morphology than is available in a 2D image. Understanding changes due to growth and treatment in 3D requires a method of 3D superimposition. Extending the traditional superimposition approach derived from 2D cephalograms to 3D structures is challenging. Because the classic Björk’s reference structures have only been evaluated in the lateral view, the stability of these structures in the transverse dimension is unknown [3]. To date, the only 3D-voxel-based regional superimposition method for the maxilla is that described by Ruellas et al [1]. They compared the cropped maxillary bone region (MAX) and the “Björk -inspired” palate and infrazygomatic region of reference (PIZ) for the reproducibility of the maxillary voxel-based superimposition. However, the suggested two regions lack the rigorous empirical evidence of Björk’s system. Different reference systems may result in different interpretations of amount and direction of jaw growth and tooth movement. Therefore, a 3D superimposition based on objective measurements rather than using specific speculated regions may provide a more valid and reproducible method of studying maxillary change and tooth movement over time.

The purpose of this study was to locate natural reference structures (NRS) in the maxilla that can be reliably used for maxillary regional superimposition in 3D. We further tested the applicability of the proposed superimposition method by assessing the concordance of this approach with Björk’s ‘stable reference structures’ in lateral projection.

## Materials and methods

### Patients

Patients who were to receive orthodontic treatment were prospectively collected at Peking University School and Hospital of Stomatology from 2013 to 2016. Inclusion criteria were: (1) Adolescent patients with cervical maturation stage (CVS2-4) at the start of the treatment. (2) Class I or II malocclusion with protrusive maxillary incisors (the value of U1/PP was larger than 115.8 + 5.7˚or/and U1/SN larger than 105.7 + 6.3˚ based on the cephalometric measurements) and less than 3 mm of crowding of the dentition; (3) Indication for the extraction of bilateral maxillary first premolars and maximum anchorage control; (4) Complete permanent dentition (not considering third molars); (5) Good health with no chronic disease or disability; 5) Patients who had CBCT images obtained immediately after implant placement and at the end of anterior tooth retraction. All the CBCT scans were obtained from the same device (Newtom VGi (Quantitative Radiology, Verona, Italy) with the following settings: field of view, 15 × 15 mm^2^; 110 kV; 6.0 mA; scan time, 18 s; and the original isotropic voxel size was 0.4 mm^3^. Ethical approval for this study was obtained from the Ethics Committee of Peking University Biomedical Sciences (PKUSSIRB_OF09_v2.0). All parents and/or their legal guardian(s) signed informed consent forms before enrollment. All the procedures were followed in accordance with the Declaration of Helsinki.

### Treatment

All patients had a convex facial profile and underwent first premolar extraction treatment with mini-screws for maximum anchorage control. After initial leveling and alignment, four self-drilling mini-screws (1.6-mm diameter, 11-mm length; Ci Bei Corporation, Zhejiang, China) were placed in the maxilla for each patient under local anesthesia. Two mini-screws were inserted into the buccal inter-radicular space between the maxillary second premolar and first molar on both sides for *en masse* retraction of the anterior teeth. The other two mini-screws were placed between the canine and lateral incisor or between the lateral and central incisor on both sides either for vertical control and torque control during retraction of the anterior teeth. *En masse* retraction of the anterior teeth used the implants for anchorage.

### Validation of the mini-screws’ stability

**Fig. 1:**
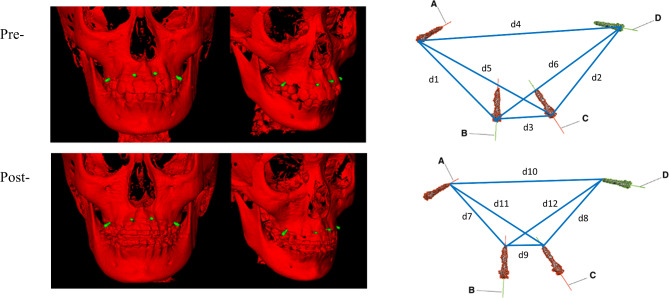
Distances measurements between the mini-screws in the maxilla

It is known that implants may be displaced by growth of the maxilla as well as orthodontic forces. It was therefore essential to evaluate the stability of the implants before they could be used as valid reference markers. The validation of implant stability was carried out by measuring the distances between the mini-screws of the maxilla, and then comparing the distances between the pairs of serial CBCT radiographs (Fig. 1). This was measured using InVivo TM 6.0 Dental software (Anatomage, San Jose, CA). The distances for joining both the head and the tail of each mini-screw were calculated, resulting in 12 linear distances for the pre-and post-images. These distances were compared using paired-t test. At P < 0.05, the difference was considered significant. Two examiners performed the measurements twice, at least 2 days in-between. Intra-examiner agreement was calculated with Intraclass Correlation Coefficient (ICC) using SPSS statistical software (version 21.0; IBM, Armonk, NY).

### Identification of potential natural reference structures (NRS)

After verifying the stability of the mini-screws, the mini-screws could be used as stable reference structures.

It was hoped that systematic analysis of serial CBCTs of subjects with these stable references might reveal additional natural reference structures (NRS) that could be used to supplement, or substitute for, the mini-screws where they were not present.

Essentially, the maxilla was represented by thousands of voxels on CBCT images. During jaw growth, every voxel changes its position. The mini-screws could be considered ‘stable anchors’. The displacement of each voxel relative to the mini-screws represented the amount of growth in this area. Collectively, when a cluster of voxels changed less, they could be considered a stable region. Detailed mathematical and technical description for the methods was as below. The image processing was performed by custom written code in MATLAB.

### 1) Initial rigid registration for superimposing the mini-screws

**Fig. 2:**
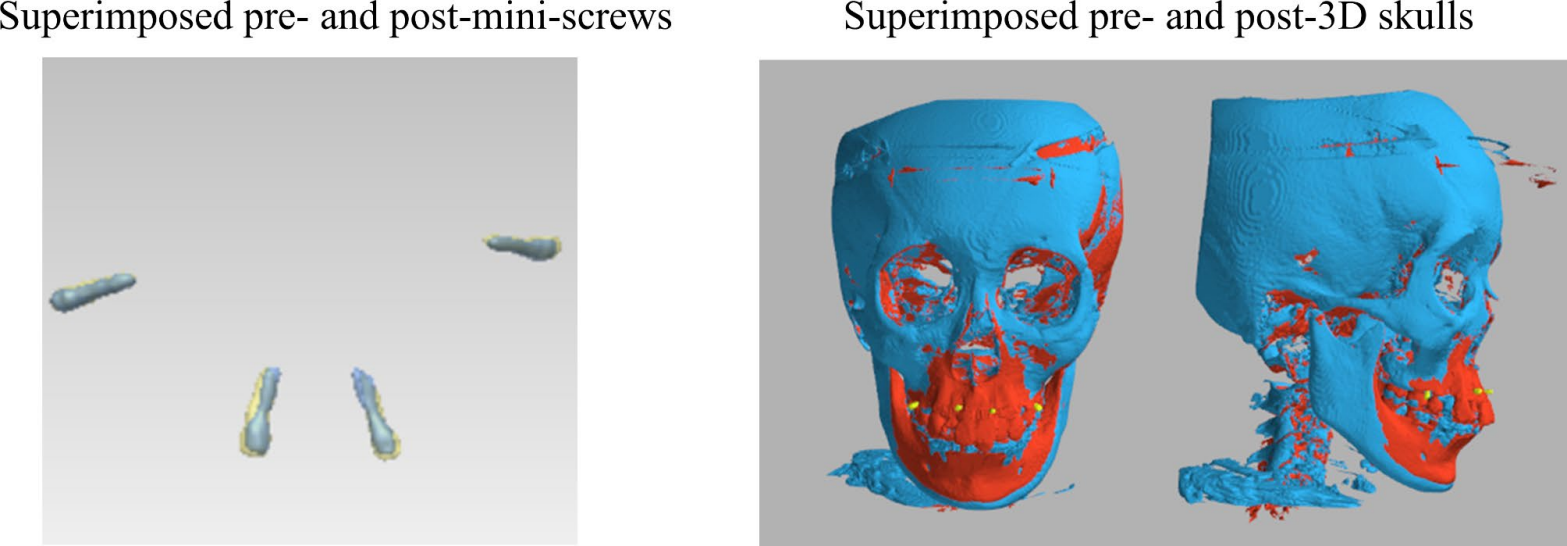
Rigid registration: Superimposition of the pre-and post-skull with the mini-screws

The images of each pair of radiographs were superimposed on 4 stable mini-screws. For each mini-screw, we annotated 2 landmarks, the head and the tip. We applied rigid transformation by translating and rotating the CBCT images until the 8 landmarks on the paired images matched (Fig. 2). The overlapping of the mini-screws was used as the initial position for the following non-rigid registration procedure.

### 2) Non-rigid registration for calculating the displacement vectors for each voxel

**Fig. 3:**
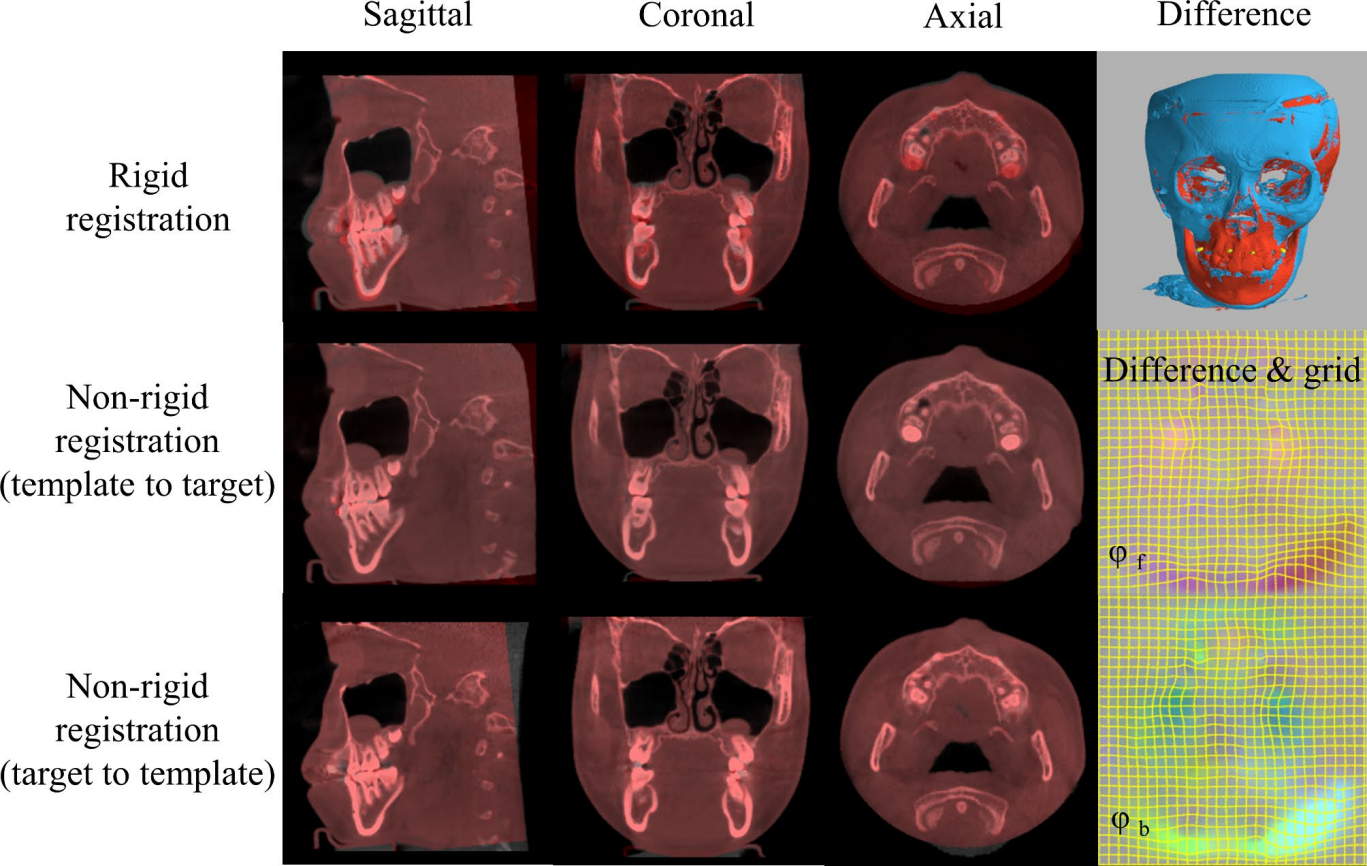
Overlapping of the template (grey) and the target CBCT image (red) before and after registration in the axial, coronal, and sagittal views. Right: the template and the target before and after registration. Rigid registration matches the template and target with the mini-screws; Non-rigid registration allows each voxel to deform until the pre- and post-treatment images match precisely. φ_f_: difference between template to target (forward registration); φ_b_: difference between target to template (forward registration)

Then non-rigid registration (deformable registration) was performed between the paired pre- and post- treatment images to evaluate the voxel displacement for the rest of the structure. Deformable registration or ‘warping’ refers to those matching techniques that involve the computation of a deformation map (grid) between points of correspondence. Correspondence means that each voxel corresponds to the same anatomical location on a given CBCT image as on all the other images, which included each pair of pre- and post-treatment volumes. The displacement vector of each voxel in each pair of pre- and post-treatment CBCT images was calculated, which represented the changes of the site during the growth and treatment. We performed the deformable registration of each pair of CBCTs by the diffeomorphic SyN registration [4] using the open-source Advanced Normalization Tools (ANTs). The code is available at http://stnava.github.io/ANTs/. This algorithm found a spatiotemporal mapping (φ ∈ Diff_0_) between the image pair so that the pre- and post-treatment images were precisely matched (Fig. 3).

### 3) Identification of potentially stable sites

The potential NRS were then mathematically and statistically detected across the entire maxillary voxels. As the displacement vectors were not comparable from patient to patient, we transferred the voxel displacement from each patient’s maxilla to a shared maxillary template to get the voxel-to-voxel correspondence. The maxillary template contains 85,325 voxels with 172*192*192 dimensions (x, y, z axes). We calculated the mean and standard deviation of the displacement among all voxels in the maxillary template. We set the grading standard according to the mean value and standard deviation, resulting in four degrees of stability. The most stable regions were selected as the NRS for the maxilla in 3D. The directional changes for each voxel were visualized as a vector map in which an inward arrow indicated morphological changes presumed to be bone resorption, and outward arrows bone deposition.

### Test of the applicability of the proposed NRS for maxillary superimposition in 3D

To test the applicability of maxillary superimposition using the stable regions identified, another dataset of longitudinal CBCT scans was used to test the proposed superimposition methods. Sample size calculation for intraclass correlation coefficients was performed with a minimally acceptable level of reliability (H0:r0) set at 0.8, with the alternative hypothesis H1:r1 set at 0.95, with α = 0.05 and power (1-β) of 0.80. With these parameters, a sample of 15 subjects was needed to be evaluated by 2 examiners. We assessed whether aligning the maxilla with proposed NRS led to concordance with the maxillary internal reference structures superimposition in the traditional 2D lateral view as suggested by Björk. This was quantitively assessed by comparing the mean sagittal and vertical tooth movements on the left and right sides measured by Björk reference structures superimposition and the proposed NRS superimposition on the 3D skulls.

**Fig. 4:**
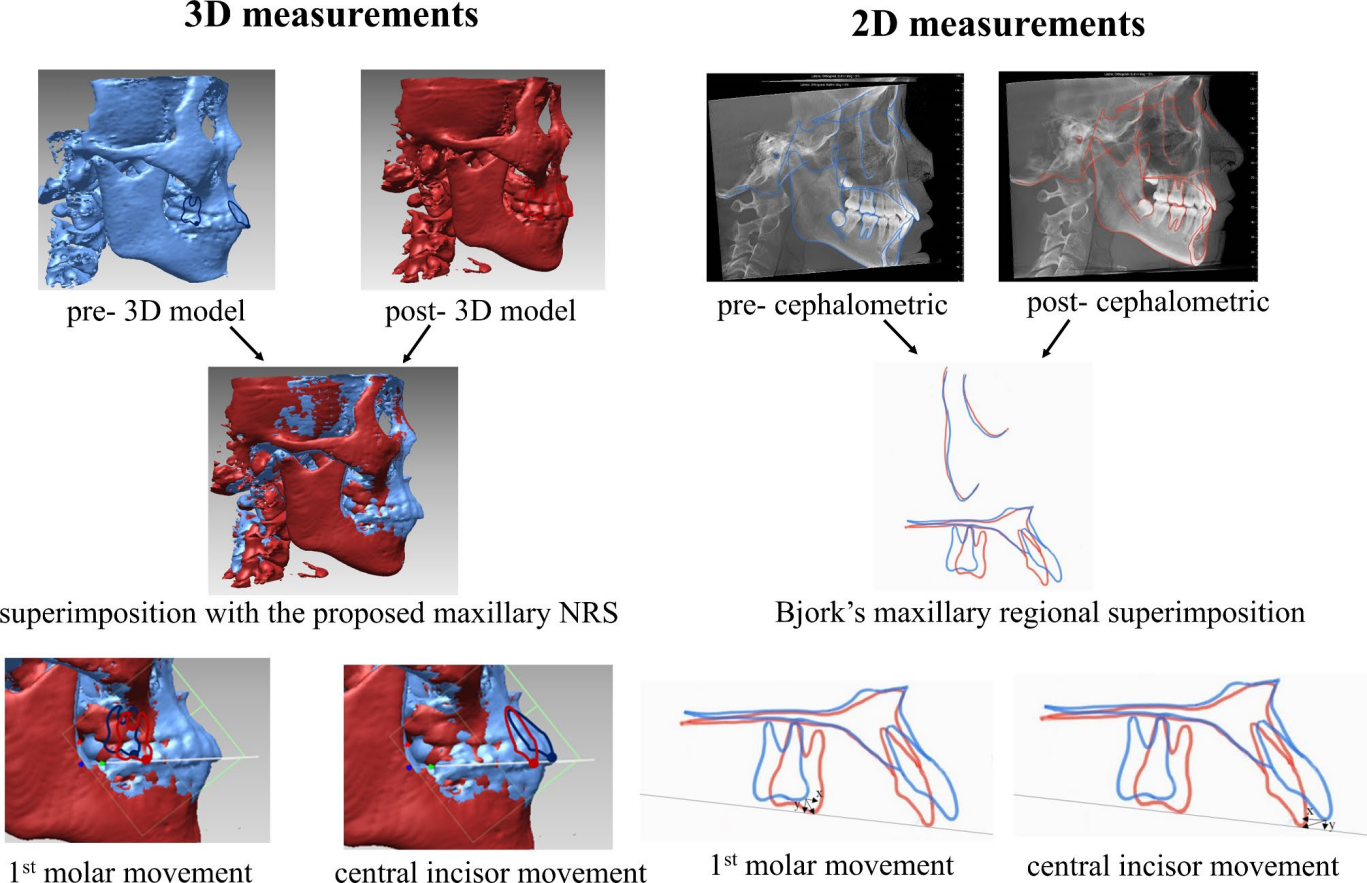
Comparison of the tooth movement measurements with the two superimposition approaches

Briefly, the mesio-buccal cusps of the maxillary molars and the midpoints of the central incisor edges were marked on reconstructed skulls from T1 and T2 CBCT images as well as CBCT-generated cephalograms (Dolphin software, version 11.7, Dolphin Imaging and Management Solutions, Chatsworth, Calif). For CBCT measurements, the NRS for each image was automatically defined by reversed mapping of the NRS from the template to each test image via previously described non-rigid registration [4]. 3D CBCT images of T1 and T2 images were superimposed using the NRS by custom analysis scripts in MATLAB. Commercially available software (such as Dolphin and OnDemand 3D) [5, 6] provides voxel-based superimposition opinions that only allows for ‘Selection of the region of reference with regular shape (such as a rectangular box)’. The natural reference structures detected in this study are irregular anatomical meaningful regions, therefore, custom writing programming is needed to locate this region and perform the superimposition. The analysis is fully automatic and therefore inherently highly reproducible. For the 2D measurements, left and right lateral cephalometric X-Rays were created using a single side of the volume. These were generated utilizing the “Build X-Rays Tool” in Dolphin 3D [7]. Then, the pre- and post-treatment cephalograms were superimposed for each side by aligning the anterior contour of the zygomatic process as suggested by Björk [2 A coordinate system, modified from a previous report by Chen et al [8] was used to measure the displacement of the maxillary right molar and left central incisor in both sagittal and vertical directions. The workflow is illustrated in Fig. 4. Two independent observers performed the analysis twice. The mean value of the tooth movement was calculated and compared between the two superimposition methods using the intraclass correlation coefficient (ICC), absolute agreement at a 95% confidence interval. All statistical computations were performed with SPSS statistical software (version 21.0; IBM, Armonk, NY).

## Results

### Patients and images

For detecting the NRS for maxillary superimposition, fifteen adolescent patients (4 males and 11 females) were originally included. Among the 15 patients, three patients had the mini-screws’ position adjusted due to treatment requirement. Two patients lost one mini-screw each, and were therefore excluded from this study. Therefore, paired images from 10 patients (3 males and 7 females) were eligible for further analyses. The CBCT images for these patients had been obtained immediately after implant placement (T1: 12.9 ± 0.8 yrs) and at the end of anterior tooth retraction (T2: 14.8 ± 0.7 yrs, time interval: 1.9 ± 0.7yrs). The demographic information for the patients was listed in Table 1.

**Table 1:** The demographic information for the patients with four mini-screws inserted in the maxilla

No.	Gender	Image T1 (yrs)	Image T2 (yrs)	Cervical vertebral stage (CVS)
1	M	12.9	14.7	3
2	M	13.0	14	2
3	M	12.6	14.1	2
4	F	13.1	16.5	4
5	F	13.3	15.6	4
6	F	13.2	14.5	3
7	F	11.6	13.0	3
8	F	11.9	14.4	2
9	F	13.2	14.8	3
10	F	12.6	14.6	3

### Validation of the mini-screws’ stability

Two examiners measured the distances among the mini-screws of the maxilla. The intraclass correlation coefficient (ICC) were 0.974 (min:0.890, max:0.999), indicating a high degree of agreement between examiners. The average distances for the mini-screws of the maxilla between the two examiners were compared between the pairs of pre- and post- treatment CBCT radiographs. There was no statistically difference among the 12 inter-mini-screw distances between the pre- and post-treatment radiographs (Table 2), which indicates that these mini-screws can be used as stationary reference points for following research.

**Table 2:** Inter-mini-screw distances comparison between pre- and post- treatment

Variable	Mean (mm)	SD (mm)	t	p-value
d1_pre - d1_post	-0.249	0.594	-1.325	0.218
d2_pre - d2_post	-0.135	1.065	-0.393	0.703
d3_pre - d3_post	-0.391	0.608	-2.031	0.073
d4_pre - d4_post	-0.342	0.489	-2.213	0.054
d5_pre - d5_post	-0.278	0.946	-0.929	0.337
d6_pre - d6_post	-0.421	0.674	-1.379	0.201
d7_pre - d7_post	-0.019	0.425	0.145	0.888
d8_pre - d8_post	-0.446	0.907	-1.554	0.155
d9_pre - d9_post	-0.205	0.674	-0.964	0.360
d10_pre - d10_post	0.665	0.404	0.328	0.750
d11_pre - d11_post	-0.474	1.021	-1.467	0.177
d12_pre - d12_post	1.545	0.741	0.659	0.527

### Identification of potentially stable sites

**Fig. 5:**
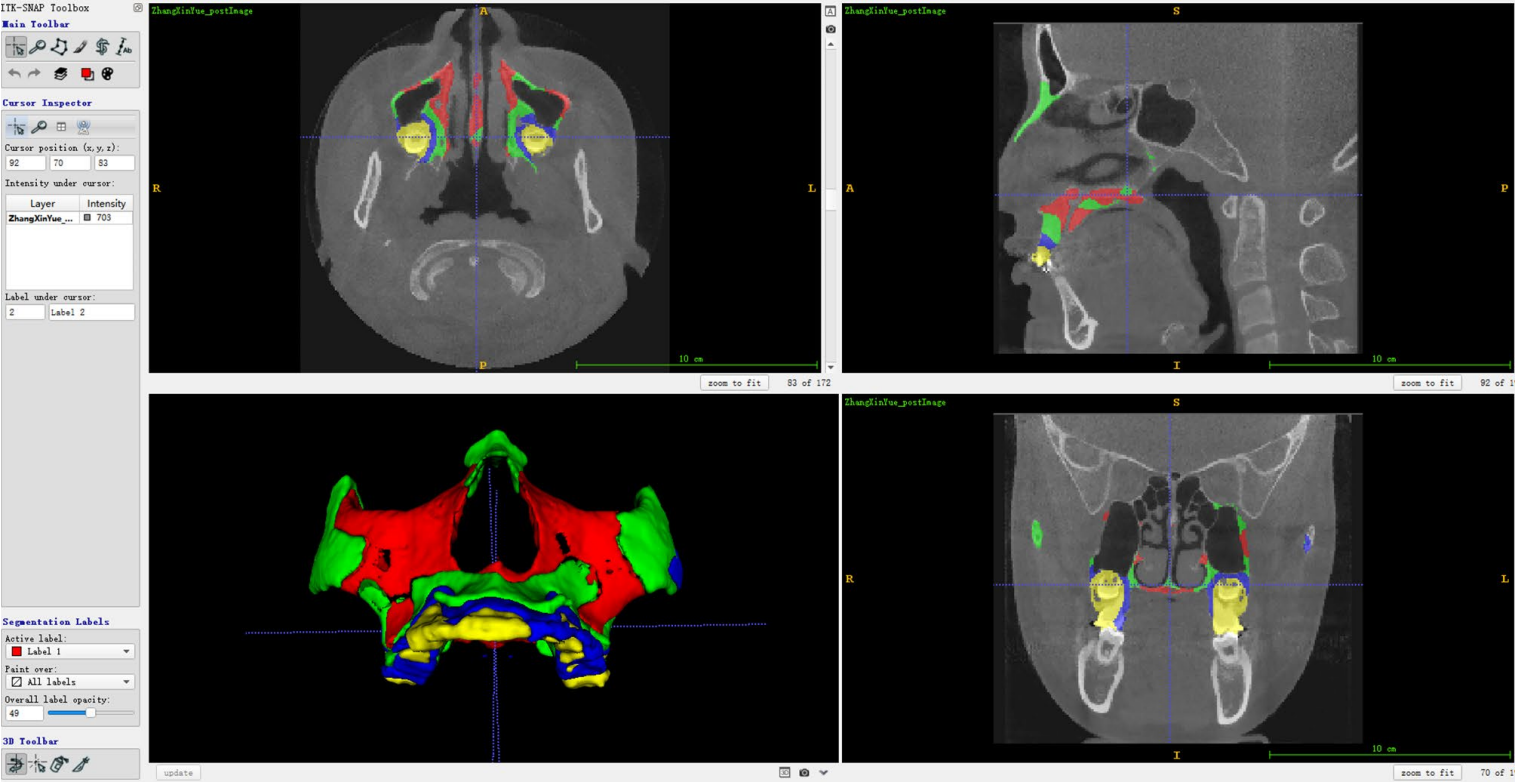
Categorization of maxillary stability according to the voxel displacement relative to the stable mini-screws

By measuring the voxel-based displacements between the pre- and post-treatment images, the mean displacement of maxillary voxels was 1.80 mm, the standard deviation (SD) was 0.60 mm. The stability of the maxilla was categorized into 4 levels: Color red indicated relatively stable regions (voxel displacement less than or equal to 1.2 mm (mean-1SD); Color green indicated less stable regions (voxel displacement from 1.2 to 1.8 mm (mean-1SD -mean); Color blue represented those voxels displaced from 1.8 to 2.4 mm (mean + 1SD). Figure 5 shows a randomly selected multi-planar image including coronal, sagittal, axial, and the 3D reconstruction view.

**Fig. 6:**
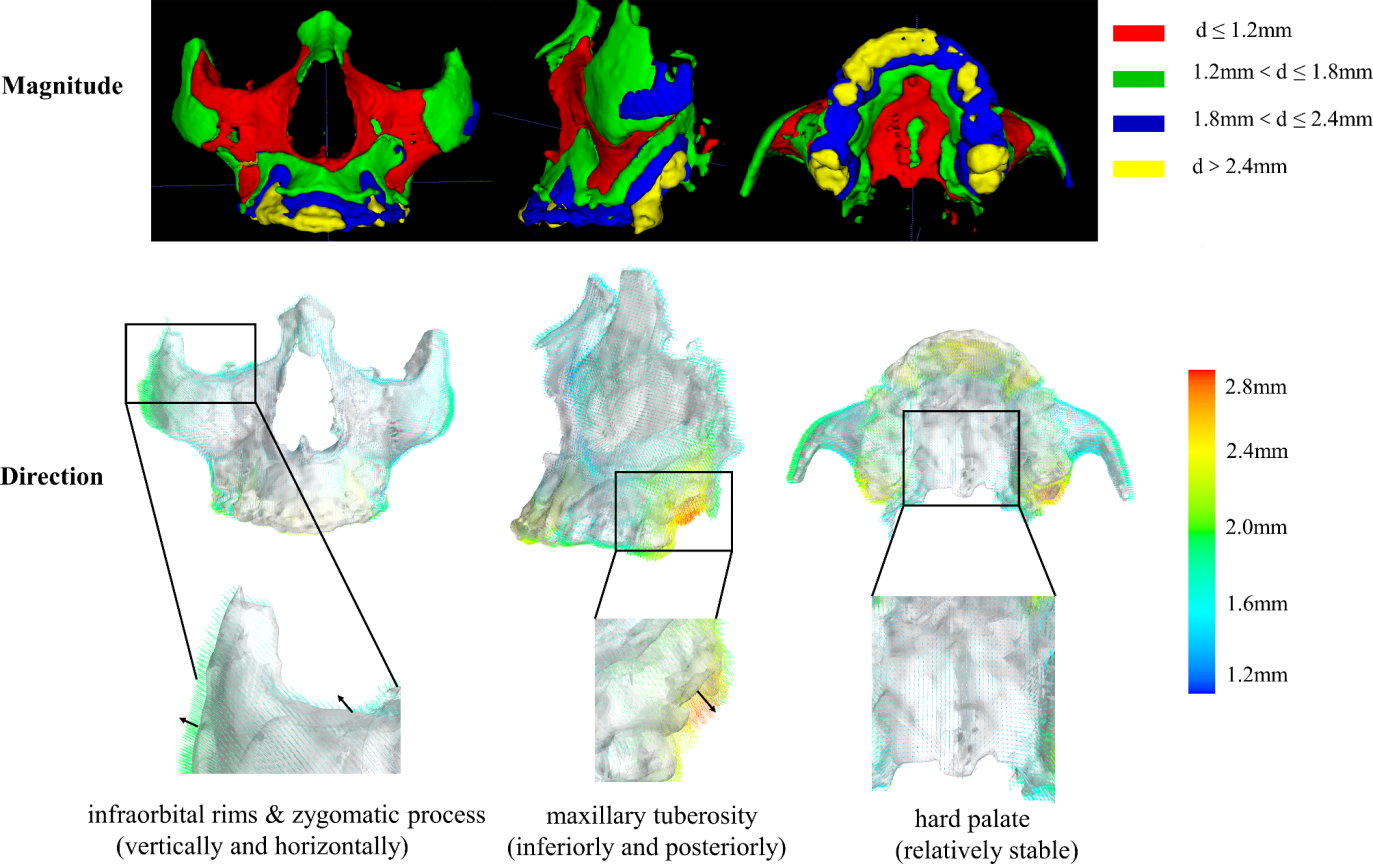
The magnitude and direction changes of each voxel of the maxilla

According to these criteria, the infraorbital rims, maxilla around the piriform foramen, the infrazygomatic crest and the hard palate (palatal vault more than 1 cm posterior to incisal foramen except the palatal suture) were found to be relatively stable, as shown in red in Fig. 6.

The directional changes for each voxel were visualized along the maxillary surface. The surface remodeling during the observation period was seen in the regions of the infraorbital rims and each side of the zygomatic process, with surface deposition around 2 mm vertically and horizontally. Approximately 2.5-3 mm elongation was seen in the maxillary tuberosity along its long axis inferiorly and posteriorly. The hard palate showed minor surface remodelling (Fig. 6).

### Concordance with Björk’s implant study

Another 15 longitudinal CBCT scans of growing patients (T1: 12.2 ± 0.63 yrs, T2: 15.2 ± 0.96 yrs) were used to further test the applicability of the proposed superimposition method by assessing the concordance of this approach with Björk’s ‘stable reference structures’ in lateral projection. Tooth movement comparison between the proposed NRS method and the classic Björk maxillary regional superimposition method was shown in Table 3. The sagittal and vertical displacements of the maxillary central incisor and maxillary first molar measured by the two superimposition methods showed good-excellent agreement.

**Table 3:** Comparison and absolute agreement of displacement of upper first molars (RU6, LU6) and upper central incisors (RU1, LU1) between the proposed natural reference structure (NRS) superimposition and the Björk’s maxillary superimposition with Intraclass Correlation (ICC)

Variable	3D Mean (SD) (mm)	2D Mean (SD) (mm)	ICC	ICC 95% CI Lower- Upper	Agreement
RU6-x	2.155(2.332)	2.122(2.579)	0.979	0.930–0.993	Excellent
RU6-y	-1.560(0.735)	-1.780(0.670)	0.875	0.591–0.962	Good
LU6-x	2.457 (1.162)	2.289 (1.098)	0.923	0.756–0.976	Excellent
LU6-y	-1.168 (1.123)	-1.137 (1.064)	0.893	0.644–0.968	Good
RU1-x	-1.930 (2.124)	-2.210 (1.874)	0.924	0.756–0.976	Excellent
RU1-y	-1.627 (1.403)	-2.077 (1.274)	0.880	0.588–0.964	Good
LU1-x	-2.116 (1.787)	-2.286 (2.233)	0.938	0.802–0.981	Excellent
LU1-y	-1.896 (1.496)	-1.980 (1.532)	0.951	0.804–0.985	Excellent

## Discussion

In this study, we detected the natural reference structures for maxillary regional superimposition of growing children in 3D. We have confirmed that the infraorbital rims, maxilla around the piriform foramen, the infrazygomatic crest and the hard palate (palatal vault more than 1 cm posterior to incisal foramen except the palatal suture) were stable during growth. These stable structures generate comparable results to Björk superimposition in the lateral view and could be used for maxillary superimposition in 3D.

Understanding the growth pattern of jaws and evaluating the amount of tooth movement in adolescent patients before and after orthodontic treatment is important. At present, the implant method and the derived stable structure method are still highly recommended for cephalometric superimposition. A lateral view of the skull is a two-dimensional image, with the attendant problems of magnification and overlap of left and right structures [9].

At the end of the 20th century, low-dose CBCT appeared, which has the potential to be a reliable method for three-dimensional evaluation of cranio-maxillofacial changes before and after orthodontic treatment [10–12]. Such a method, however, would rely on the identification of biologically stable structures in 3 dimensions. The method of identifying stable structures performed in the classic studies of Björk using tantalum implants in growing subjects cannot be replicated in 3D because of the ethical concerns around subjecting healthy growing children to the higher radiation doses required for spiral/ helical CT or CBCT examination. Another approach is required.

In the end of 20th century, Kawamata et al. first applied voxel-based superimposition on orthognathic surgery patients [13]. Later, Cevidanes et al. further developed methods for voxel-based superimpositions on growing and nongrowing dental patients in 3 dimensions [14–16]. Comparable stable structures for the upper and lower jaw have not yet been clarified. In the existing literature, maxillary and mandibular regional superimposition has been performed using the same philosophy as 2D cephalometrics, which is to define stable anatomic landmarks or structures and then align these landmarks sequentially for ‘best-fit’ of said stable structures. Two problems exist. First, those anatomical structures that are considered stable on 2D radiographs during growth may not be so in 3D. For example, the mandibular canal shifts laterally during growth which is not seen on 2D cephalometric images [3], the validity of the proposed stable structures remains untested. Ruellas et al. provided the only evidence in the literature for maxillary superimposition in 3D [1]. They used the cropped maxillary and the “Björk inspired” palate and infrazygomatic region as stable regions. These regions showed similar results and adequate interobserver and interobserver reproducibility in maxillary superimposition. However, the exact method by which these structures were identified and the details of how their stability was verified has not been published. Accurate superimposition methodology is a prerequisite for meaningful assessment of skeletal and dental positional changes [17, 18]. The lack of robust methods for assessing jaw growth in 3D may account for the large number of controversial issues and unanswered questions surrounding clinical treatment modalities for craniofacial growth evaluation.

Therefore, in this study we verified the stability of the mini-screws and then used them as stationary references to detect potential NRS of the maxilla. Our results showed the infraorbital rims, maxilla around the piriform foramen, the infrazygomatic crest and the hard palate (palatal vault that 1 cm distal to incisor foramen except the palatal suture) were stable during growth. These anatomical structures could act as substitutes for the mini-screws when they are missing in the CBCT images. Superimposition of two maxillae could therefore be achieved by rotating and translating one CBCT onto another until the cumulative distance between these stable regions is minimized.

Detection of natural reference structures in CBCT images using stable bone plate and screws as references was pioneered by Nguyen et al [19]. They evaluated reliability of mandibular registrations using the chin and symphysis and confirmed that these regions were stable for 3D mandibular regional superimposition. The regions they chose incorporated prior guesswork about their stability and overlooked other potential internal stable regions within the jaw. Springate et al. conducted a systematic search for stable regions in the mandible in children with tantalum implants on lateral cephalometric radiographs [20]. Their search and match procedures were carried out automatically by computer using the mathematical procedure known as cross-correlation. However, the overlay of the left and right structures hindered the accuracy for detected regions.

Inspired by both studies we utilized a more sophisticated stable regions detection strategy on CBCT images. Without relying on pre-defined stable regions, we assumed that there may be many previously unreported NRS and that they could be widely distributed throughout the maxilla. Through the non-linear registration, the spatial information between pairs of images was registered and the displacement for each voxel of the maxilla was objectively calculated. All the voxels that belonging to the maxilla formed a ‘deformation map’ during growth and treatment. Then the deformed grid could be objectively evaluated, and the stability of the maxillary internal structures could be objectively categorised.

In this study, the maxilla at piriform aperture level was detected as a stable region. This occupies a similar area to that suggested by Ruellas et al [1]. The infrazygomatic crest was also found to be relatively stable during the observation period. This are which used to be known as the ‘key ridge’ provides a broad buttress of bone on which the upper first molar rests [20]. However, as described in the ABO video of maxillary superimposition, they do not recommend superimposition onto the key ridge which remodels downward and the orbital rim which remodels upwards. It is possible that ABO’s recommendation is based on long-term follow up of the Bjork’s sample as his study described the growth of the maxilla from the age of 4 years until adulthood. Our observation period is less than Bjork’s study. Therefore, it is likely that the infrazygomatic crest, key ridge area and infraorbital rims are relatively stable during the 2–3 year period, but become unstable on long-term follow up. The palatal region was found to be stable 1 cm distal to incisor foramen, which is in agreement with Chen et al. who found that the medial 2 ⁄3 of the third rugae and the regional palatal vault dorsal to the third rugae were stable regions in adult patients [8]. The growth of the palatal suture was clearly visualized. This area showed an approximately 1.2–1.4 mm displacement during the observation period. This finding was in agreement with Björk’s study that the growth in the median suture, measured between the lateral implants was on average 1 mm per year during puberty [2]. Nevertheless, it is important to recognize that no structure within a living subject can be totally stable, at least at the ultrastructural or cellular levels [21]. From a practical perspective, if the level of instability of a reference structure is smaller than clinical detectable value, then this cannot be distinguished from genuine stability.

Our validation test confirms that the displacements of the maxillary central incisor and maxillary first molar, measured by the two superimposition methods, show no significant difference, confirming that the proposed NRS method for maxillary superimposition approximates the classic Björk type cephalometric superimposition. In addition, the morphological changes we have observed in the maxilla are consistent with the known bone remodeling pattern described by Enlow and Hans. For example, the sagittal lengthening of the bony maxillary arch is produced by posterior remodeling of the maxillary tuberosity [22]. The infrazygomatic crest (key ridge) is stable during growth, which also indirectly confirms that this region is safer for extra-radicular bone screws during full arch distalization. Most importantly, our evaluation goes beyond the histological approach in the cadaver to living patients by visualizing not only the location of bone remodeling, but also quantifying the magnitude and direction of the changes. This possibly provides individualized, far-reaching insight into maxillary growth with a strong biological basis.

In this study conclusions are limited in that all the cases have been treated orthodontically, and it is difficult to separate the normal growth of the maxilla from the intervention. The time intervals for T1 and T2 images are not long enough to unveil the full picture of maxillary grow in 3D. Untreated growth samples and longer follow-up are needed to evaluate usefulness of the proposed method. Another limitation is that the automatic detection of the NRS based on the non-rigid registration is not a clinician friendly technique, which requires basic knowledge of coding. Our ultimate goal is to make an open source software with a user-friendly interphase for maxilla regional superimposition in 3D.

## Conclusions

In this study, we detected the natural reference structures for the maxilla in growing patients by using a modified regional implant method, the infraorbital rims, maxilla around the piriform foramen, the infrazygomatic crest and the hard palate (palatal vault more than 1 cm posterior to incisal foramen except the palatal suture) were identified as stable. These regions can be used to assess morphological changes in the maxilla over time. We have validated the method against the gold standard method of Björk’s maxillary regional superimposition and shown it to give results consistent with the known biology of the growth of the maxilla. Not only does this approach offer the opportunity to improve understanding of maxillary growth in 3D but can be applied directly in the clinical setting.

## Data Availability

The datasets used during the current study are available from the corresponding author on reasonable request.

## List of abbreviations

3D: three-dimensional
2D: two-dimensional
NRS: natural reference structures
CT: computed tomography
CBCT: cone-beam computed tomography
CVS: cervical vertebral stage
